# Impact of Janus kinase inhibitors and methotrexate on interstitial lung disease in rheumatoid arthritis patients

**DOI:** 10.3389/fimmu.2024.1501146

**Published:** 2024-12-16

**Authors:** Shota Kurushima, Tomohiro Koga, Masataka Umeda, Naoki Iwamoto, Ritsuko Miyashita, Takatomo Tokito, Daisuke Okuno, Hirokazu Yura, Hiroshi Ishimoto, Takashi Kido, Noriho Sakamoto, Yukitaka Ueki, Hiroshi Mukae, Atsushi Kawakami

**Affiliations:** ^1^ Department of Immunology and Rheumatology, Division of Advanced Preventive Medical Sciences, Nagasaki University Graduate School of Biomedical Sciences, Nagasaki, Japan; ^2^ Department of Respiratory Medicine, Nagasaki University Graduate School of Biomedical Sciences, Nagasaki, Japan; ^3^ Rheumatic Disease Centre, Sasebo Chuo Hospital, Sasebo, Japan

**Keywords:** rheumatoid arthritis, interstitial lung disease, RA-ILD, JAK inhibitors, methotrexate, epithelial-mesenchymal transition

## Abstract

**Objectives:**

Little is known about how various treatments impact the progression of interstitial lung disease (ILD) in rheumatoid arthritis (RA) patients. Here, we compared ILD progression in RA patients treated with Janus kinase inhibitors (JAKi) or biological disease-modifying anti-rheumatic drugs (bDMARDs). *In vitro* experiments were also performed to evaluate the potential effects of the drugs on epithelial–mesenchymal transition (EMT), a key event in pulmonary fibrosis.

**Methods:**

This retrospective study included 93 RA-ILD patients who initiated treatment with JAKi, tumour necrosis factor inhibitors (TNFi), or abatacept between 2017 and 2020. Worsening ILD was quantified by changes in chest computed tomography (CT) scans between baseline and follow-up (mean 14 months, range 6–51 months). Response to treatment was evaluated using Disease Activity Score-28 with erythrocyte sedimentation rate (DAS28-ESR). Expression of the EMT marker N-cadherin in A549 lung cells was assessed by western blotting.

**Results and discussion:**

Worsening ILD was detected in 19.4% (7/36), 16.7% (5/30), and 22.2% (6/27) of patients treated with JAKi, abatacept, and TNFi, respectively. Multivariate analysis identified female gender (P=0.043) and >10% fibrotic lesions (P=0.015) as significant predictors of worsening ILD. DAS28-ESR-based non-responder status was also significantly associated with worsening ILD (P=0.0085). In vitro, combination treatment with methotrexate and baricitinib significantly impeded EMT progression. Worsening ILD was associated with more extensive fibrotic lesions at baseline and female gender in RA patients treated with JAKi or bDMARDs. JAKi and methotrexate co-treatment may prove beneficial in modifying key events underlying the pathogenesis of RA-ILD.

## Introduction

Rheumatoid arthritis (RA) is an inflammatory autoimmune disease characterized by chronic joint inflammation, and it is often accompanied by extra-articular effects, such as interstitial lung disease (ILD). RA-associated ILD (RA-ILD) is a significant complication and can affect prognosis (1, 2). Although the advent of biological disease-modifying anti-rheumatic drugs (bDMARDs) and Janus kinase inhibitors (JAKi) has expanded the treatment options for RA, the optimal treatment of RA-ILD remains undefined (3, 4). Notably, several studies have highlighted the need to be aware of the risks of respiratory infection and drug-induced lung injury when using anti-rheumatic drugs in RA patients with ILD (5, 6).

Currently, abatacept, a fusion protein of the extracellular domain of cytotoxic T lymphocyte-associated protein 4 (CTLA4) and the Fc region of human IgG1, is considered to be the most reasonable option for treating RA patients with ILD (7); however, recent reports suggest that the efficacy and safety of JAKi may be comparable to those of abatacept in terms of their impact on the disease behavior of RA-ILD (8, 9). Factors involved in the progression or acute exacerbation of RA-ILD include usual interstitial pneumonia (UIP) pattern, decreased forced vital capacity, cigarette smoking, and high titers of anti-cyclic citrullinated protein antibody (ACPA) (10, 11). Additionally, risk factors associated with new-onset RA-ILD include older age, male gender, cigarette smoking, high titers of rheumatoid factor and ACPA, and poor control of arthritis activity (12–14).

The epithelial–mesenchymal transition (EMT) is a key physiological process during which epithelial cells lose their polarity and change to mesenchymal phenotypes. Downregulation of the epithelial cell marker E-cadherin and upregulation of the mesenchymal marker N-cadherin, also known as cadherin switching, are characteristic features of EMT (15, 16). Although of great physiological importance, EMT is also associated with various pathological states, especially after cell injury and chronic inflammation (17). Indeed, EMT is considered to be one of the key processes involved in the pathogenesis of RA-ILD, similar to the events leading to idiopathic pulmonary fibrosis (18). *In vitro* studies with human alveolar type II cells have shown that EMT can be induced by treatment with factors such as transforming growth factor-β and interleukin (IL)-6, and it has been reported to be inhibited by blocking the JAK/STAT signaling pathway (19). However, the effects of methotrexate (MTX), an anchor drug for managing RA (20), on EMT remain largely unexplored.

The aim of the present study was to compare the temporal changes in chest computed tomography (CT) images of patients with RA-ILD treated with JAKi or bDMARDs and to identify factors associated with deterioration of RA-ILD on imaging. In addition, we investigated the possible mechanism of action of JAKi and MTX treatment on the fibrotic state of RA-ILD patients by investigating their effects on IL-6-induced EMT in alveolar epithelial cells *in vitro*. Our results shed light on the potential for JAKi and MTX therapy to inhibit the progression of RA-ILD.

## Materials and methods

### Study design and participants

**Figure 1:**
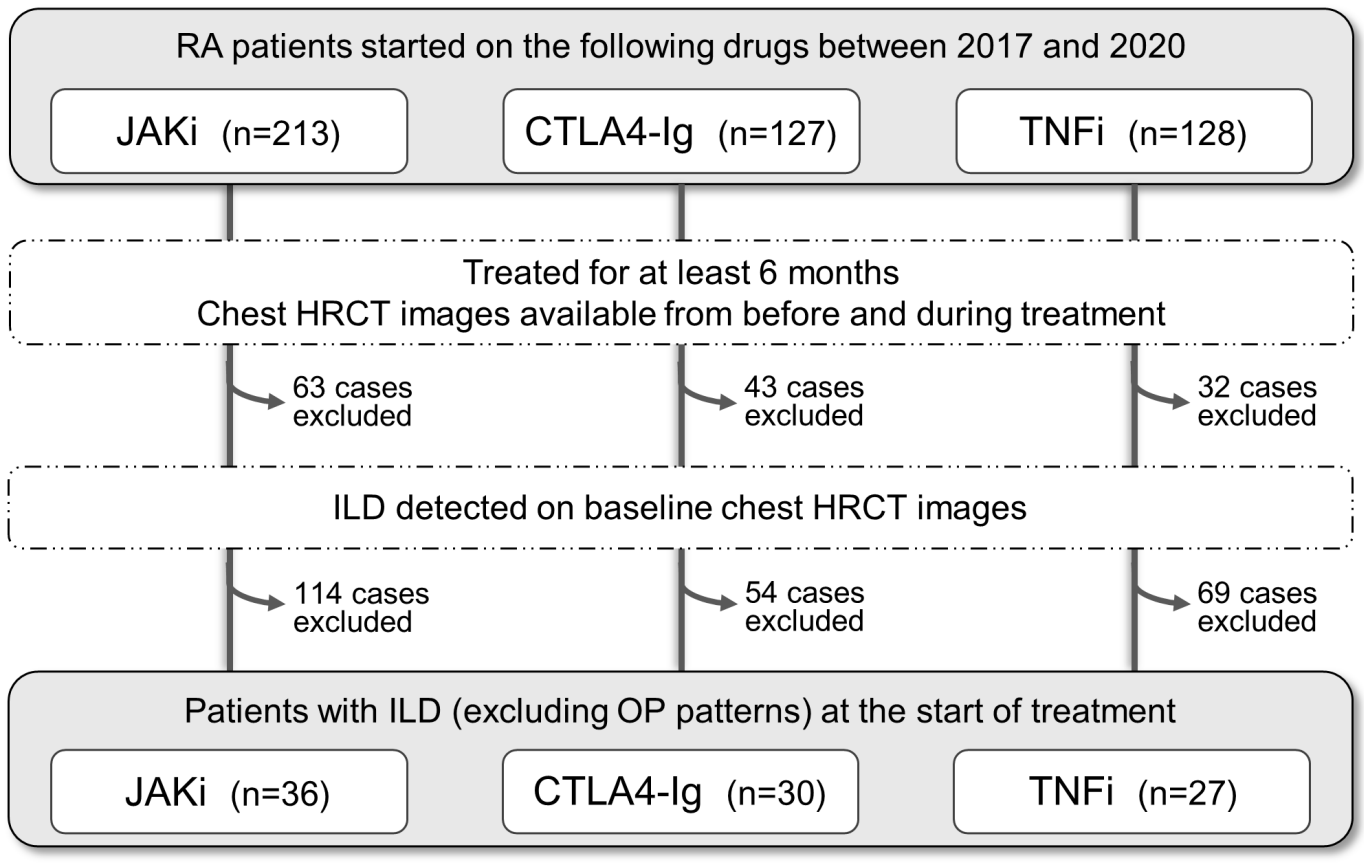
Selection of rheumatoid arthritis patients with interstitial lung disease for this study. Flowchart of the selection process. The initial pool comprised total 468 patients with rheumatoid arthritis (RA) who started treatment with JAK inhibitors (JAKi), T cell co-stimulation inhibitor (CTLA4-Ig), or TNF inhibitors (TNFi) between 2017 and 2020. Patients were excluded if (i) treatment duration was <6 months, (ii) chest high-resolution computed tomography (HRCT) images were unavailable at either baseline or follow-up, (iii) interstitial lung disease was not noted on baseline HRCT images, (iv) bronchiolitis or emphysema was the main lesion, and (v) organizing pneumonia (OP) pattern was diagnosed.

This retrospective cohort study focused on RA patients with ILD who started a new treatment program with JAKi (tofacitinib, baricitinib) or bDMARDs (TNFi, abatacept) at Nagasaki University Hospital or Sasebo Chuo Hospital between January 2017 and December 2020. A flow diagram illustrating the selection of research subjects is shown in **Figure 1**. From the initial pool of 468 included patients, those who discontinued these drugs within 6 months of initiation or whose pre- and post-treatment chest CT images were not available were excluded (n=138). The remaining 330 patients were assessed for ILD using high-resolution CT (HRCT) imaging at the start of treatment. Patients with bronchiolitis or emphysema as the main lesion and those diagnosed with organizing pneumonia were excluded. The final group of 93 patients with confirmed ILD were selected for analysis.

### Ethics statement

This study was approved by the Institutional Review Board of Nagasaki University (approval number 11032819), and informed consent was obtained from each patient.

### Chest CT image evaluation and scoring

The classification of HRCT patterns of RA-ILD was performed by experienced pulmonologists affiliated with the Japanese Respiratory Society (RM and TT), and by board-certified members (DO, HY, HI, TK, and NS) according to the joint statement of the American Thoracic Society/European Respiratory Society/Japanese Respiratory Society/Latin American Thoracic Society (21). The patterns were evaluated by two pulmonologists. In case of disagreement, one of the board-certified pulmonologists acted as a third reviewer and made the final assessment. The presence of ground-glass attenuation, reticular shadows, consolidation, traction bronchiectasis/bronchiolectasis and honeycombing, and the proportion of each lesion in the total lung field were assessed on a case-by-case basis by two pulmonologists independently. Ground-glass attenuation, reticular shadows, and consolidation were defined as inflammatory lesions; and traction bronchiectasis/bronchiolectasis and honeycombing were defined as fibrotic lesions. A CT score was calculated using the formula described by Ichikado et al. (22).

CT score = (1 × percentage of lung area with normal attenuation) + (2 × percentage of lung area with ground-glass attenuation or reticular shadows) + (3 × percentage of lung area with consolidation) + (4 × percentage of lung area with ground-glass attenuation or reticular shadows accompanied by traction bronchiectasis/bronchiolectasis) + (5 × percentage of lung area with consolidation accompanied by traction bronchiectasis/bronchiolectasis) + (6 × percentage of lung area with honeycombing).

In this formula, the percentage represents the proportion of the total lung area affected. For example, if the whole lung was normal, the score would be 1 × 100 = 100 points. If 80% of the lung was normal and 20% was affected by honeycombing, the score would be calculated as (1 × 80) + (6 × 20) = 200 points. The final score was obtained by averaging the scores of the two evaluators. Cases were deemed “worsened ILD” if the scores assigned by both evaluators were higher post-treatment compared with pre-treatment.

### Disease activity and improvement assessment

Disease activity status was evaluated using the 4-variable Disease Activity Score in 28 joints with erythrocyte sedimentation rate (DAS28-ESR). The definitions were: high disease activity (HDA) >5.1, moderate disease activity (MDA) ≥3.2 to ≤5.1, low disease activity (LDA) ≥2.6 to <3.2, and remission <2.6 (23). Patients were assigned to responder groups using the European League Against Rheumatism (EULAR) response criteria based on DAS28-ESR scores: good responders (improvement >1.2 and current score ≤3.2), moderate responders (improvement >0.6 to ≤1.2 and current score ≤5.1 *or* improvement >1.2 and current score >3.2), and non-responders (improvement ≤0.6 regardless of current score *or* improvement >0.6 to ≤1.2 and current score >5.1) (24).

### Cell culture

A549 cells (human type II alveolar epithelial cell line) were purchased from the European Collection of Authenticated Cell Cultures (ECACC, #86012804) and cultured in Kaighn’s modified Ham’s F12 medium (F12K Medium, Life Technologies, Tokyo, Japan) supplemented with 10% fetal bovine serum and 1% penicillin/streptomycin. Cells were maintained at 37°C in a 5% CO_2_ environment. For experiments, cells were plated into 6-well plates at 2.0 × 10^5^ cells/well and treated with MTX (FUJIFILM Wako, Osaka, Japan) and/or baricitinib (Chemscene, Monmouth Junction, NJ, USA) at the indicated concentrations for 48 h and 3 h, respectively. EMT was induced by addition of IL-6 (Sigma-Aldrich, St. Louis, MO, USA) at 50 ng/mL for 24 h (25) and the cells were harvested for analysis.

### Western blot analysis

Proteins were extracted from A549 cells using RIPA Buffer (FUJIFILM Wako) supplemented with protease inhibitors (Takara Bio, Shiga, Japan) and phosphatase inhibitors (Thermo Fisher Scientific, Waltham, MA, USA). Lysates were centrifuged at 15,000 rpm for 15 min at 4°C and the supernatants were collected. N-cadherin and β-actin levels were quantified using an automated Wes Simple Western system (ProteinSimple, San Jose, CA, USA) according to the manufacturer’s instructions. The primary antibodies were anti-N-cadherin (#GTX127345, GeneTex, Irvine, CA, USA; 1:100 dilution) and anti-β-actin (#GTX109639, GeneTex; 1:500 dilution).

### Statistical analysis

Continuous variables are presented as medians with interquartile ranges (IQR), and categorical variables as numbers and percentages. Statistical analyses were performed using JMP Pro 17 software (SAS Institute, Cary, NC, USA). Proportions of binary data were compared using Fisher’s exact test. Continuous variables were compared using either Student’s *t*-test or the Mann–Whitney U test. Predictive factors for ILD progression on CT images were identified using Cox regression analysis, with variable selection based on existing knowledge, clinical experience, and the results of univariate analyses. Statistical significance was defined as a two-tailed P value of <0.05. This study was exploratory and no adjustment for multiplicity was planned.

## Results

### Characteristics of participants

Patient characteristics at baseline are summarized in **Table 1**. This study included 36 patients treated with JAKi (29 with tofacitinib, 7 with baricitinib), 30 patients treated with the T cell co-stimulation inhibitor CTLA4-Ig (abatacept), and 27 patients treated with TNFi (14 with golimumab, 6 with etanercept, 4 with adalimumab, and 3 with certolizumab pegol) (**Table 2**). A total of 61 of the 93 patients (66%) were female. The median age was 71 years (IQR 66–78 years) and the median duration of RA was 9 years (range 3–15 years). The rheumatoid factor positivity rate was 95% and the ACPA positivity rate was 91%. The proportion of patients taking concomitant MTX and glucocorticoids (GC) was 38% and 55%, respectively. The median baseline DAS28-ESR was 4.85 (IQR 3.88–5.55). HRCT imaging assessment classified 30 patients (32%) as having definite UIP pattern and the remaining 63 patients (68%) as having non-UIP pattern, which included probable UIP, indeterminant for UIP, and alternative diagnosis pattern (21). Baseline RA disease activity was highest in the JAKi-treated group; the proportion of UIP pattern was highest in the CTLA4-Ig-treated group; and the proportion of concomitant treatment with MTX was highest in the TNFi-treated group.

**Table 1:** Demographic and clinical characteristics of RA patients by treatment group.

Variable	All Patients (n=93)	JAKi Group (n=36)	CTLA4-Ig Group (n=30)	TNFi Group (n=27)
Female, n (%)	61 (66%)	27 (75%)	17 (57%)	17 (63%)
Age, years, median (IQR)	71 (66–78)	69 (65–78)	71 (67–78)	73 (66–78)
RA duration, years, median (IQR)	9 (3–15)	9 (6–14)	8 (2–14)	8 (2–17)
RF positive, n (%)	88 (95%)	32 (89%)	29 (97%)	27 (100%)
ACPA positive, n (%)	85 (91%)	32 (89%)	28 (93%)	25 (93%)
Concomitant use of MTX, n (%)	35 (38%)	14 (39%)	7 (23%)	14 (52%)
Concomitant use of GC, n (%)	51 (55%)	22 (61%)	20 (67%)	9 (33%)
DAS28-ESR, median (IQR)	4.85 (3.88–5.55)	5.19 (4.39–5.93)	4.90 (3.52–5.46)	4.54 (3.40–5.18)
CT pattern, n	UIP 30, non-UIP 63	UIP 6, non-UIP 30	UIP 15, non-UIP 15	UIP 9, non-UIP 18
Extent of fibrotic lesions >10%, n (%)	21 (23%)	6 (17%)	9 (30%)	6 (22%)
CT score, median (IQR)	122 (109–147)	110 (106–132)	126 (112–153)	129 (114–145)

ACPA, anti-citrullinated protein antibody; CT, computed tomography; CTLA4-Ig, T cell co-stimulation inhibitor; DAS28-ESR, Disease Activity Score based on a 28-joint count–erythrocyte sedimentation rate; GC, glucocorticoids; IQR, interquartile range; JAKi, Janus kinase inhibitor; MTX, methotrexate; RA, rheumatoid arthritis; RF, rheumatoid factor; TNFi, tumor necrosis factor inhibitor; UIP, usual interstitial pneumonia.

**Table 2:** Multivariate analysis of factors associated with worsening CT score in RA-ILD patients.

Variable	Worsening (n=18)	Not Worsening (n=75)	P Value	Multivariate Hazard Ratio (95% Confidence Interval)	P Value
Treatment			0.87		
JAKi, n	7	29			
CTLA4-Ig, n	5	25			
TNFi, n	6	21			
Gender			0.10	3.76 (1.05–13.52)	0.043
Female/Male, n	15/3	46/29			
Age			0.12	2.15 (0.69–6.69)	0.19
≥75/<75 years, n	10/8	26/49			
RA Onset Age			0.059	1.32 (0.43–4.05)	0.63
≥65/<65 years, n	11/7	26/49			
RA Duration			0.80		
≥10 years/<10 years, n	9/9	34/41			
RF Status			0.58		
Positive/Negative, n	18/0	70/5			
ACPA Status			0.35		
Positive/Negative, n	18/0	67/8			
Concomitant Use of MTX			0.013	0.19 (0.03–1.08)	0.061
Yes/No, n	2/16	33/42			
Concomitant Use of GC			0.43		
Yes/No, n	8/10	43/32			
DAS28-ESR			0.61		
High disease activity, n	6	30			
Moderate disease activity, n	7	37			
Low disease activity, n	1	4			
Remission, n	2	3			
CT Pattern			0.58		
UIP/non-UIP, n	7/11	23/52			
Extent of Fibrotic Lesions			0.0040	3.51 (1.28–9.63)	0.015
>10%/≤10%, n	9/9	12/63			

ACPA, anti-citrullinated protein antibody; CT, computed tomography; CTLA4-Ig, T cell co-stimulation inhibitor; DAS28-ESR, Disease Activity Score based on a 28-joint count–erythrocyte sedimentation rate; GC, glucocorticoids; ILD, interstitial lung disease; IQR, interquartile range; JAKi, Janus kinase inhibitor; MTX, methotrexate; RA, rheumatoid arthritis; RF, rheumatoid factor; TNFi, tumor necrosis factor inhibitor; UIP, usual interstitial pneumonia.

### CT score evaluation of chest CT images

CT scores were calculated at baseline and follow-up (average, 14 months; range, 6–51 months). An increase in CT score from baseline to follow-up (indicating worsening lung lesions) was observed in 18 of the 93 patients (19.4%); which included 19.4% (7/36) of the JAKi group, 16.7% (5/30) of the CTLA4-Ig group, and 22.2% (6/27) of the TNFi group (**Table 2**).

### Association between disease activity and worsening CT score

**Figure 2:**
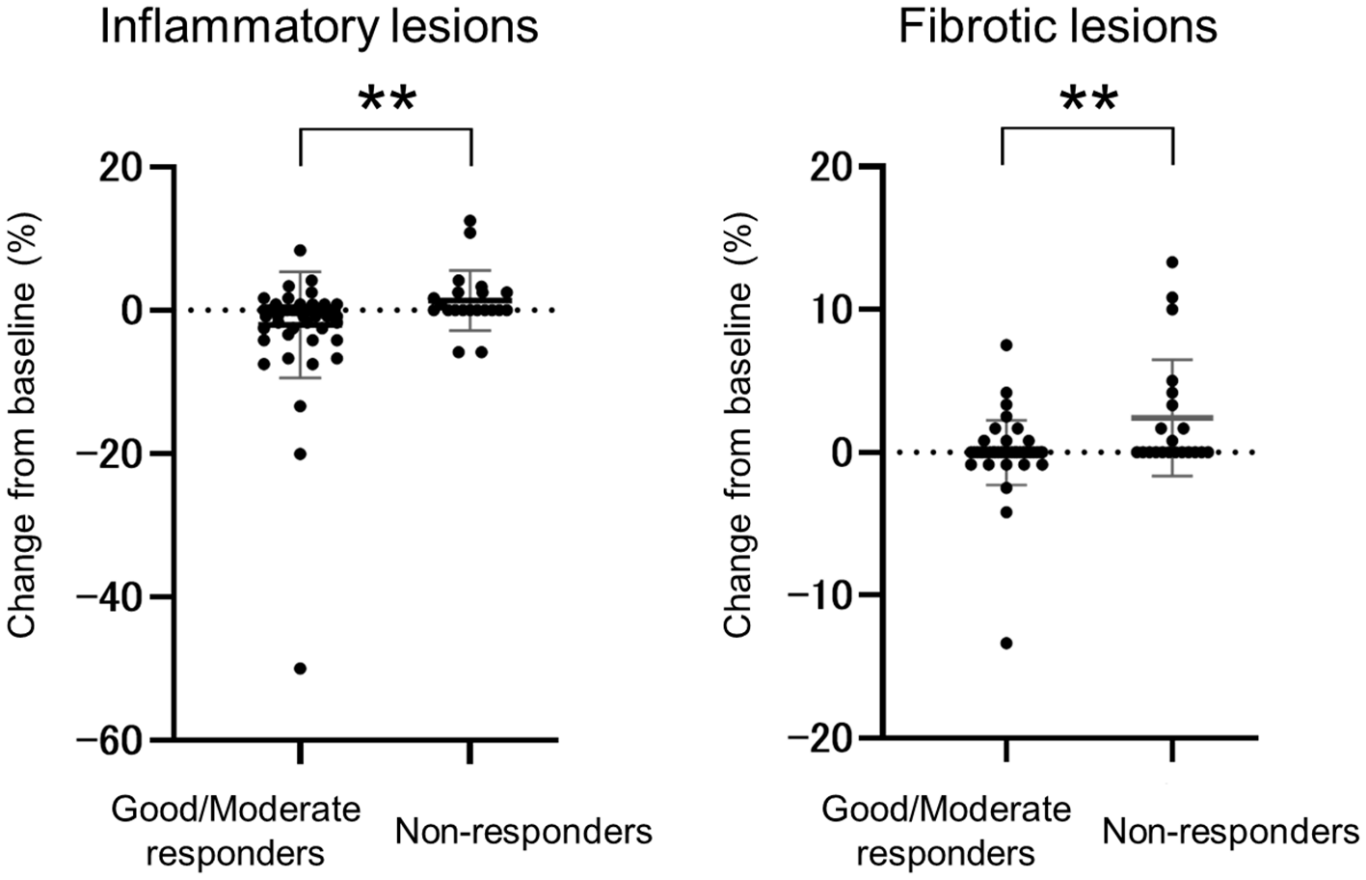
Comparison of inflammatory and fibrotic lesion changes between good/moderate responders and non-responders. Change from baseline to follow-up in the good/moderate responder group (n=58) and non-responder group (n=21) based on EULAR response criteria and DAS28-ESR scores. Data are presented as the mean and standard deviation; circles represent individual patients. ** P <0.01.

The relation between disease activity and worsening CT score was investigated in a total of 79 patients with continuously assessed DAS28-ESR. At baseline, 40%, 48%, 6%, and 6% of patients had HDA, MDA, LDA, or were in remission, respectively; at follow-up, these proportions were 8%, 49%, 25%, and 18%, respectively. The percentage of patients with worsening CT scores was 17.7% (6/34) of patients with LDA or in remission compared with 22.2% (10/45) of patients with HDA or MDA (P=0.78). Based on the EULAR response criteria, non-responders had a significantly higher rate of CT score worsening (42.9%, 9/21) compared to the good/moderate responders (12.1%, 7/58) (P=0.0085) at follow-up. When the changes in inflammatory lesions and fibrotic lesions were examined separately, both were found to be significantly worse in the non-responders compared with the good/moderate responders (P=0.0050 for inflammatory lesions, P=0.0024 for fibrotic lesions) (**Figure 2**).

### Analysis of predictive factors for CT score worsening

**Table 2** presents the results of Cox multivariate analysis for the 93 patients according to worsening (n=18) or not worsening (n=75) CT scores at follow-up. Explanatory variables were selected based on their potential for involvement in the progression of RA-ILD as assessed by existing knowledge, clinical experience, and the results of the univariate analysis. Multivariate analysis identified “extent of fibrotic lesions >10%” (HR 3.51, 95% CI 1.28–9.63, P=0.015) and “female gender” (HR 3.76, 95% CI 1.05–13.52, P=0.043) as factors significantly associated with an increased risk of worsening CT score. Co-administration of MTX showed a trend towards a reduced risk; however, the difference narrowly missed reaching statistical significance (HR 0.19, 95% CI 0.03–1.08, P=0.061).

**Figure 3:**
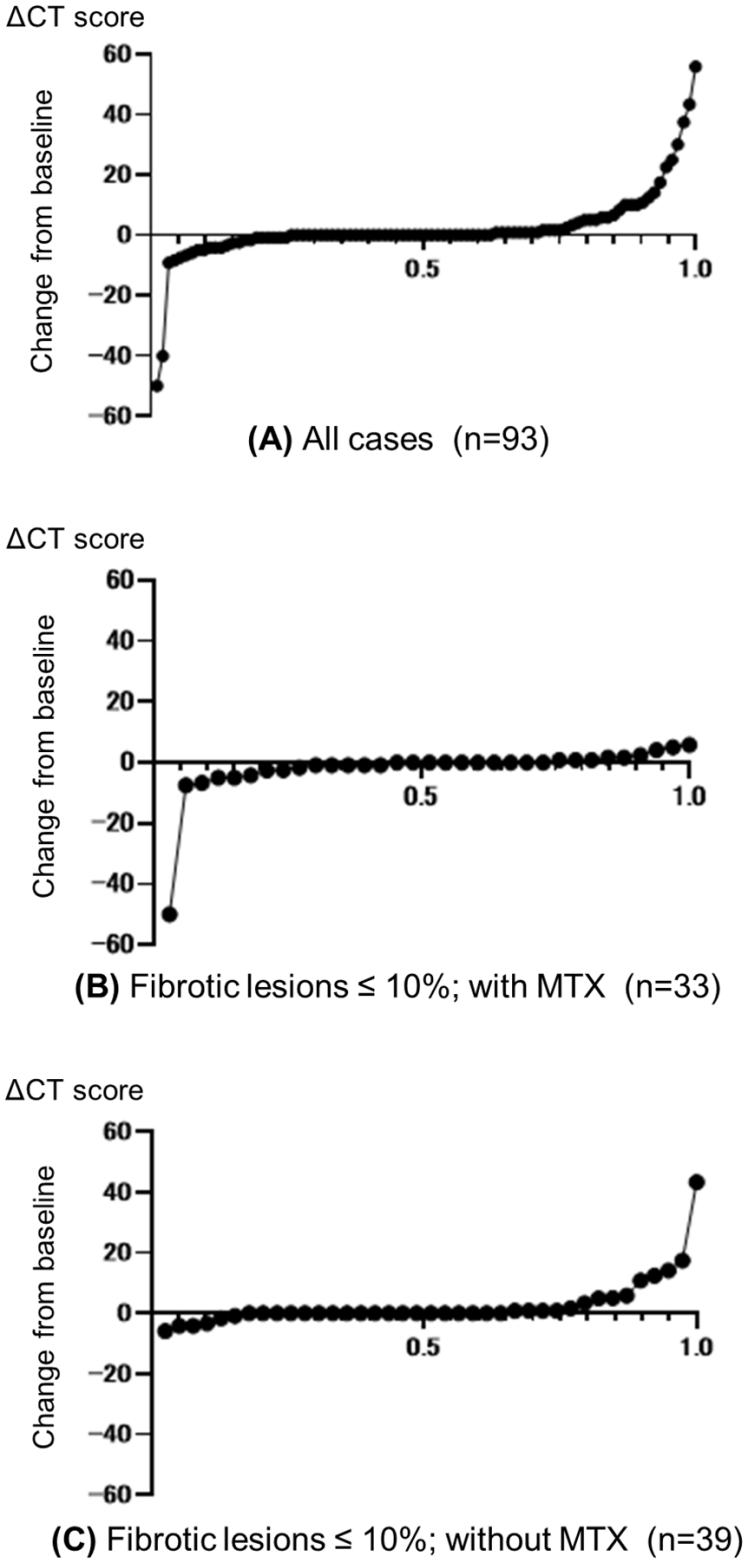
Cumulative probability plots of change from baseline in CT scores. Change from baseline to follow-up (ΔCT score) for **(A)** all patients and **(B, C)** patients with pulmonary fibrosis lesions in ≤10% of the total lung field who were treated with **(B)** or without **(C)** concomitant methotrexate (MTX). Circles represent individual patients.

The change in CT score (ΔCT score) for each patient is displayed using cumulative probability plots in **Figure 3**. The beneficial effect of MTX co-treatment was evident even among patients with fibrotic lesions in less than 10% of the lung field. Only 3% (1/33) of patients in the MTX group had worsening CT scores compared with 21% (8/39) of patients in the non-MTX group (P=0.033). Non-responders (EULAR criteria) were also significantly less frequent in the MTX group (12%, 3/25) compared with the non-MTX group (38%, 13/34) (P=0.038).

### *In vitro* effects of MTX and baricitinib on EMT of A549 cells

**Figure 4:**
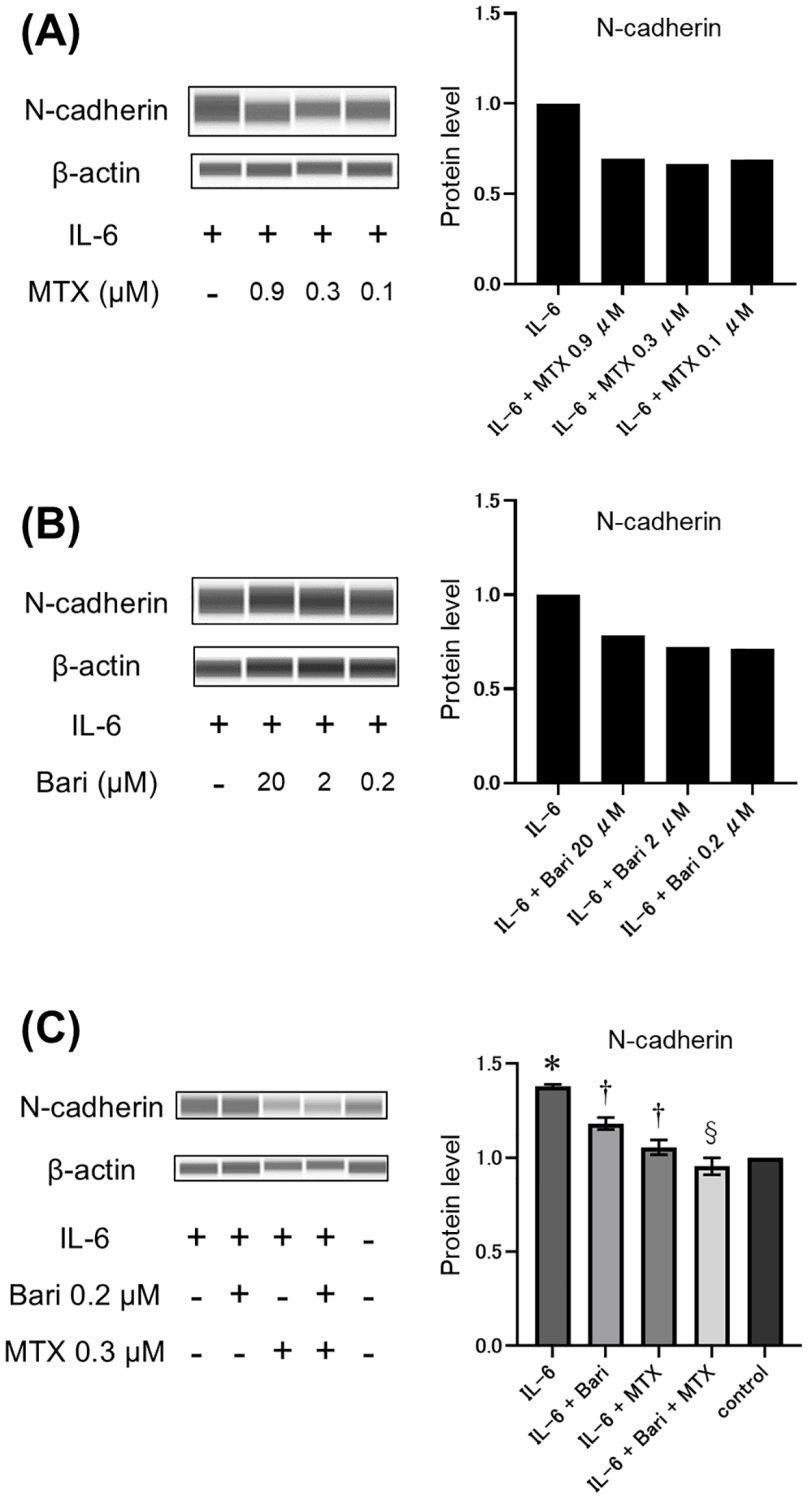
Effects of MTX and baricitinib treatment on IL-6-induced N-cadherin expression in A549 cells. **(A–C)** Western blot analysis (left panels) and relative quantification (right panels) of N-cadherin expression in A549 cells pre-treated with various concentrations of methotrexate (MTX) for 48 h **(A)**, baricitinib (Bari) for 3 h **(B)**, or 0.2 μM Bari and/or 0.3 μM MTX for 48 h and 3 h, respectively **(C)** and then stimulated with 50 ng/mL IL-6 for 24 h Data are expressed as the mean of duplicates (a and b) or the mean ± standard error of quadruplicates **(C)**. *P<0.001 compared to the control group, †P<0.01 compared to the IL-6 group, and §P<0.05 compared to the IL-6+Bari group.

Because the EMT is a crucial mechanism in the pathogenesis of RA-ILD fibrosis, we assessed the potential effects of MTX and the JAKi baricitinib on the EMT *in vitro*. To this end, western blot analysis was performed to quantify expression of the mesenchymal phenotypic marker N-cadherin in human A549 cells pre-treated with the drugs and then treated with IL-6 to induce EMT. Pre-treatment of A549 cells with either MTX for 48 h (**Figure 4A**) or baricitinib for 3 h (**Figure 4B**) inhibited the IL-6-induced increase in N-cadherin expression. However, combined pre-treatment with MTX at 0.3 µM and baricitinib at 0.2 µM, which are concentrations comparable to their maximal serum drug concentrations in clinical practice, resulted in a significant reduction in IL-6-induced N-cadherin levels that was superior to the reductions achieved by treatment with baricitinib alone (**Figure 4C**). This result suggests that MTX and baricitinib have an additive effect in modulating progression through the EMT, which is a key pathway in fibrosis pathologies.

## Discussion

In this study, we identified factors associated with worsening of pre-existing ILD in RA patients treated with JAKi or bDMARDs and, further, examined the impact of JAKi and MTX pre-treatment on IL-6-induced EMT in A549 cells *in vitro*, thereby providing new insights into therapeutic strategies for patients with RA-ILD.

We quantified changes in ILD by assigning a CT score on chest HRCT images. Overall, 19.4% of the 93 patients had worsening CT scores, which included 19.4% of the JAKi group, 16.7% of the CTLA4-Ig group, and 22.2% of the TNFi group. It should be noted that the clinical characteristics of the patients in the present study were not homogeneous between groups, which limits our ability to directly compare the effects of these drugs on RA-ILD. Abatacept is currently considered the optimal bDMARD for RA patients with ILD (7, 26); however, recent studies reported that the efficacy and safety of JAKi are comparable to those of abatacept in RA-ILD (8, 9). The proportions of patients with worsening of RA-ILD in our study were similar to those reported in previous studies, even though the HRCT assessment methods differed between studies (8, 9, 27).

In our patient population, multivariate analysis identified “extent of fibrotic lesions >10%” and “female gender” as factors significantly associated with worsening RA-ILD. Lesions with traction bronchiectasis/bronchiolectasis and honeycombing were defined as fibrotic lesions in our study. Although UIP pattern has been reported to be an exacerbating factor in RA-ILD (11), it is difficult to clearly distinguish between UIP and non-specific interstitial pneumonia on HRCT. Therefore, it is important to evaluate both the presence and the extent of fibrotic lesions to predict disease behavior in RA-ILD (10, 28). Fibrosing lung disease affecting more than 10% of the lung volume on HRCT, as used in the inclusion criteria of the INBUILD trial (29), suggests a progression-prone phenotype, serving as an indicator for the use of anti-fibrotic drugs.

We found that patients with poor improvement in DAS28-ESR (non-responders based on EULAR response criteria) showed a higher tendency for worsening CT score than good or moderate responders. This result is consistent with previous findings that high RA disease activity significantly affects RA-ILD progression (30, 31), suggesting that reducing systemic inflammation with anti-rheumatic drugs could modify the natural course of RA-ILD. Although male gender is generally reported as a risk factor for worsening RA-ILD (11), our study identified female gender as a predictor of worsening ILD based on HRCT imaging. One possible explanation for this discrepancy is that, in our cohort, a higher proportion of women than men were non-responders (32.1% vs. 15.4%). It is also possible that the results could be driven by specific characteristics of our study population, such as age, smoking status, complications, or the severity of RA at baseline.

Patients co-administered MTX exhibited a lower rate of worsening ILD in this study, which is intriguing. Consistent with guidance that MTX is contraindicated in patients with severe respiratory disorders or severe pulmonary fibrosis, as judged by chest radiographs (32), most patients receiving MTX in our cohort had ≤10% fibrotic lesions. Notably, the MTX group had better outcomes than the non-MTX group even after accounting for potential bias related to the severity of lung lesions. Moreover, the MTX group contained fewer non-responders (based on EULAR response criteria) than did the non-MTX group, suggesting that a reduction in disease activity might have contributed to modifying the natural course of RA-ILD.

The local effects of anti-rheumatic drugs in lung tissues are not well understood. Recent findings from a retrospective study of RA patients treated with adalimumab, abatacept, tocilizumab, rituximab, or tofacitinib showed that the incidence of new-onset RA-ILD was lowest in patients treated with tofacitinib (33). Furthermore, it has been reported that MTX may lead to delayed onset and a lower incidence of RA-ILD in early RA inception cohorts (34). The mechanisms by which tofacitinib and MTX inhibit the development of RA-ILD are not fully understood. We conducted *in vitro* experiments with A549 cells to determine the effect of baricitinib and MTX on EMT, a key mechanism in lung fibrosis. Our finding that baricitinib attenuated IL-6-induced EMT in A549 cells corroborates the findings of a previous study (25). However, in contrast to reports that MTX induces EMT-like changes in A549 cells (35), we found that MTX inhibited IL-6-induced EMT. Interestingly, the effect on EMT of baricitinib and MTX in combination was greater than that of baricitinib alone, suggesting that combining JAKi and MTX may prove beneficial in modifying the pathogenesis of lung fibrosis. Similar results were obtained in the RA-BEGIN trial, which showed that the baricitinib and MTX combination therapy group had the lowest rate of progression of structural joint damage on X-ray (36). A meta-analysis of JAKi and MTX therapy also indicated that combination MTX and JAKi therapy was superior to JAKi monotherapy in achieving LDA or remission in RA patients, although it also carried a higher risk of adverse events (37). The beneficial effect of JAKi and MTX on RA disease activity may also aid in slowing the progression of ILD. Further studies will be required to elucidate the detailed mechanisms involved; however, our findings suggest the possibility that JAKi and MTX may have a combined effect on fibrosis in local lung tissue.

This study has several limitations. First, the retrospective design limits the ability to infer causal relationships between treatments and observed outcomes; thus, prospective studies are warranted to confirm these findings. Second, the small sample size of 93 cases did not allow us to apply propensity score matching to adjust for imbalances in clinical characteristics or to perform subgroup analyses according to specific drug types. Third, the lack of information on smoking status and diabetes diagnosis prevented us from analyzing the impact of these risk factors on RA-ILD progression. Fourth, it was not possible to analyze biomarkers such as Krebs von den Lungen-6, which indicates disease activity in ILD. Finally, the interpretation of HRCT images, which is critical for the classification of fibrosis patterns, was dependent on clinician assessment and thus subject to inter-observer variability, potentially introducing bias in the evaluation of ILD progression.

In conclusion, we found that extensive fibrotic lesions, female gender, and poor improvement in disease activity were significantly associated with worsening ILD in RA patients treated with JAKi and bDMARDs, and we also showed the potential for co-administered MTX to modify the course of RA-ILD in these patients. Additionally, our *in vitro* results suggest that the combination of MTX and JAKi may have an additive effect in attenuating EMT, a key mechanism in lung fibrosis. Further research will be necessary to fully understand these interactions and to refine treatment strategies for RA-ILD.

## Data Availability

The raw data supporting the conclusions of this article will be made available by the authors, without undue reservation.
